# Prevalence of Plasmid-Mediated Quinolone Resistance and Aminoglycoside Resistance Determinants among Carbapeneme Non-Susceptible *Enterobacter cloacae*

**DOI:** 10.1371/journal.pone.0047636

**Published:** 2012-10-23

**Authors:** Shifeng Huang, Wei Dai, Shan Sun, Xiaojiao Zhang, Liping Zhang

**Affiliations:** Department of Laboratory Medicine, the First Affiliated Hospital of Chongqing Medical University, Chongqing, China; Charité-University Medicine Berlin, Germany

## Abstract

**Background:**

Simultaneous resistance to aminoglycosides and fluoroquinolones in carbapeneme non-susceptible (CNS) isolates will inevitably create problems. The present study was performed to characterize the prevalence of the plasmid-mediated quinolone resistance determinants (*QRDs*) and aminoglycoside resistance determinants (*ARDs*) among the CNS *Enterobacter cloacae* (*E. cloacae*) isolates in a Chinese teaching hospital, and to acquire their molecular epidemiological characteristics.

**Methods:**

The β-lactamases genes (including class A carbapenemase genes *bla_KPC_* and *bla_SME_*, metallo-β-lactamase genes (MBLs) *bla_IMP_*, *bla_VIM_* and *bla_NDM_*, and extended spectrum β-lactamases (ESBLs),*bla_CTX-M_*, *bla_TEM_* and *bla_SHV_*), QRDs (including *qnrA*, *qnrB*, *qnrS* and *aac(6′)-Ib-cr*) and ARDs (including *aac(6′)-Ib*, *armA* and *rmtB*) of these 35 isolates were determined by PCR and sequenced bidirectionally. The clonal relatedness was investigated by pulsed-field gel electrophoresis (PFGE).

**Results:**

Of the 35 isolates, 9 (25.7%) harbored a carbapenemase gene; 23 (65.7%) carried ESBLs; 24 (68.6%) were QRD positive; and 27 (77.1%) were ARD positive. Among the 5 *bla_IMP-8_* positive strains, 4 (80%) contained both ESBL and *QRD* genes, and all the 5 (100%) harbored *ARD* genes. Of the 23 ESBLs positive isolates, 6 (26.1%) were carbapenemase positive, 14 (60.9%) were QRD positive, and 18 (78.3%) were ARD positive. PFGE revealed genetic diversity among the 35 isolates, indicating that the high prevalence of CNS *E. cloacae* isolates was not caused by clonal dissemination.

**Conclusion:**

QRD and ARD genes were highly prevalent among the CNS *E. cloacae* isolates. Multiple resistant genes were co-expressed in the same isolates. The CNS *E. cloacae* isolate co-expressing *bla_NDM-1_*, *bla_IMP-26_*, *qnrA1* and *qnrS1* was first reported.

## Introduction

*Enterobacter cloacae (E. cloacae)* has recently emerged as an important hospital pathogen [1]. Increasingly reported extended-spectrum β-lactamases (ESBLs) and carbapenemases in *E. cloacae* represent an emerging public health concern [2]. The vast majority of ESBLs belong to the TEM-, SHV- and CTX-M-type enzymes [3]. Since ESBL-producing bacteria are often multidrug resistant (MDR), carbapenems represent one of the therapeutic options of last resort for life-threatening infections due to these organisms [4]. Although several mechanisms of carbapenem resistance have been reported, most of the mechanisms are related to the spread of plasmid-mediated acquired carbapenemases belonging to Ambler class A (KPCs) and class B (VIMs, IMPs, and NDM-1) β-lactamases [5]. These enzymes compromise the clinical efficacy of almost the whole armamentarium of antimicrobial drugs, leaving clinicians with only a limited number of “last-line” agents such as colistin [6]. Detection of infected patients and carriers with carbapenemase producers is therefore becoming a major health issue to prevent their spread [5].

Of note, the ESBLs and carbapenemases are often encoded by genes located on large plasmids and these also carry genes for resistance to other antimicrobial agents such as aminoglycosides [7], [8] and fluoroquinolones [8], [9]. Thus, very broad antibiotic resistance extending to multiple antibiotic classes is now a frequent characteristic of ESBL- and/or carbapenemase-producing enterobacterial isolates [1], [10].

Quinolone resistance may be conferred by plasmid-mediated quinolone resistance (PMQR) determinants, including the *qnr* genes, *qnrA*, *qnrB* and *qnrS* [11], and the recently identified *aac(6′)-Ib-cr* [12]. Although the PMQR determine relatively small increases in the MICs of quinolones, these changes are sufficient to facilitate the selection of mutants with higher levels of resistance [13], and the penetration of PMQR into the population of *Enterobacter spp*. were found to be coincided with a rapid increase in fluoroquinolone resistance [14]. More importantly, they were shown to be associated with other resistance elements, for instance, *qnr* genes were found to be co-carried with various *ESBLs* genes on the same plasmid [15], as well as with metallo-β-lactamase (MBLs) genes [16] and the class A carbapenemase gene *bla_KPC_* [17].

Aminoglycosides are an important class of antimicrobial agents for the treatment of life-threatening bacterial infections. Several mechanisms for aminoglycoside resistance have been described previously, with bacterial expression of drug-metabolizing enzymes, such as the clinically widespread aminoglycoside N-6'-acetyltransferase-Ib (AAC(6')-Ib) being the most common mechanism of resistance to aminoglycoside antibiotics, especially in Gram-negative clinical isolates [18]. Moreover, 16S rRNA methyltransferases, such as ArmA and RmtB, are especially troublesome due to their wide target range and their ability to confer high levels of resistance [19]. Of note, they were shown to be associated with other resistance elements. For instance, 16S RNA methylase genes have been demonstrated to be co-expressed with various ESBLs [20] and carbapenemases genes such as *bla_NDM-1_* [21], *bla_IMP_* [22] and *bla_KPC-2_* [23] on the same plasmid.

Of concern, both *PMAR* and *PMQR* genes are not yet being taken into account in resistance screening by clinical microbiology laboratories. However, on the other hand, analysis of the drug resistance profiles of the recent carbapenem-non-susceptible (CNS) *E. cloacae* isolates from our hospital showed that most of the CNS *E. cloacae* strains (32/35, 91.4%) were resistant to both fluoroquinolones (CIP and LEV) and aminoglycosides (GM, TOB and AK) according to the 2012 CLSI breakpoints. Simultaneous resistance to quinolone and aminoglycosides in CNS isolates will inevitably create problems, as many carbapenemase producers may carry unrelated drug-resistance genes such as the quinolone resistance determinants (*QRDs*) and aminoglycoside resistance determinants (*ARDs*), and selection pressure with structurally unrelated antibacterial drugs (for instance, aminoglycosides and fluoroquinolone) may contribute to their spread. Thus, the present study was initiated to explore the prevalence of PMQR and PMAR Determinants among the 35 CNS *E. cloacae* isolates collected from Sep 2009 to Feb 2012 in our hospital. ESBL genes, carbapenemase genes, *QRD* genes and *ARD* genes of the CNS *E. cloacae* isolates were detected to observe the complex genotypes, and all of the strains were characterized by antibiotic resistance phenotyping and pulsed-field gel electrophoresis (PFGE) to understand whether the strains were epidemiologically related.

## Materials and Methods

### Bacterial Isolates

Between September 2009 and February 2012, 986 clinical isolates of *E. cloacae*, as identified by VITEK2 compact and API system (bioMerieux, Hazelwood, MO, France) were isolated in our laboratory, 35 (3.55%) of which were non-susceptible to ertapenem (ETP) or imipenem (IMP) according to the 2012 MIC interpretive criteria recommended by the Clinical and Laboratory Standards Institute (CLSI).

### Antimicrobial Susceptibility Test

MICs of ceftazidime (CAZ), ceftriaxone (CRO), cefepime (FEP), imipenem (IMP), ertapenem (ETP), gentamycin (GM), tobramycin (TOB), amikacin (AK), ciprofloxacin (CIP) and levofloxacin (LEV) were determined for all isolates using a microbroth dilution method and interpreted based on the 2012 MIC interpretive criteria recommended by the CLSI.

### Detection and Sequencing of β-lactamases, ARD and QRD Genes

All the 35 *E. cloacae* isolates with reduced susceptibility to imipenem (MIC≥2 µg/ml) or ertapenem (MIC≥1 µg/ml) were defined as CNS isolates, which were than PCR screened using custom primers (Table 1) targeting β-lactamases genes, *QRD* and *ARD* genes. PCR amplicons were sequenced for both strands by Invitrogen (Invitrogen, Shanghai), and sequences were analyzed.

**Table 1:** PCR primers for β-lactams, quinolinones and aminoglycosides resistance genes.

Genes	Primers	Sequences (5′-3′)’)	Amplicon length(bp)
*bla_KPC_*	F	ATGTCACTGTATCGCCGTCT	892
	R	TTTTCAGAGCCTTACTGCCC	
*bla_SME_*	F	AACGGCTTCATTTTTGTTTAG	820
	R	GCTTCCGCAATAGTTTTATCA	
*bla_VIM_*	F	TTATGGAGCAGCAACGATGT	920
	R	CAAAAGTCCCGCTCCAACGA	
*bla_IMP_*	F	CATGGTTTGGTGGTTCTTGT	488
	R	ATAATTTGGCGGACTTTGGC	
*bla_NDM-1_*	1-F	CAGCACACT TCCTATCTC	292
	1-R	CCGCAACCATCCCCTCTT	
	2-F	GGCGGAATGGCTCATCACGA	287
	2-R	CGCAACACAGCCTGACTTTC	
*bla_TEM_*	F	GTGCGCGGAACCCCTATT	919
	R	TTACCAATGCTTAATCAGTGAGGC	
*bla_SHV_*	F	CTTTACTCGCCTTTATCGGC	1031
	R	TTACCGACCGGCATCTTTCC	
*bla_CTX-M-3′_*	F	AATCACTGCGCCAGTTCACGCT	479
	R	GAACGTTTCGTCTCCCAGCTGT	
*bla_CTX-M-14′_*	F	TACCGCAGATAATACGCAGGTG	355
	R	CAGCGTAGGTTCAGTGCGATCC	
*qnrA*	F	ATTTCTCACGCCAGGATTTG	413
	R	GAGATTGGCATTGCTCCAGT	
*qnrB*	F	GATCGTGAAAGCCAGAAAGG	469
	R	ACGATGCCTGGTAGTTGTCC	
*qnrS*	F	GCAAGTTCATTGAACAGGGT	428
	R	TCTAAACCGTCGAGTTCGGCG	
*aac(6′)-Ib-cr*	F	ATATGCGGATCCAATGAGCAACGCAAAAACAAAGTTAG	544
	R	ATAGCGAATTCTTAGGCATCACTG CGTGTTCGCTC	
*aac(6′)-Ib*	F	TATGAGTGGCTAAATCGAT	395
	R	CCCGCTTTCTCGTAGCA	
*armA*	F	CCGAAATGACAGTTCCTATC	846
	R	GAAAATGAGTGCCTTGGAGG	
*rmtB*	F	ATGAACATCAACGATGCCCT	769
	R	CCTTCTGATTGGCTTATCCA	
*IntI 1*	F	GGTCAAGGATCTGGATTTCG	480
	R	ACATGCGTGTAAATCATCGTC	

### Conjugation Experiment and Antimicrobial Susceptibility Test by VITEK2 Compact System

Conjugation transfer assay was performed in broth culture with *E. coli* EC600 as the recipient. Donor and recipient cells were mixed at a ratio of 1∶1. Transconjugants were selected on MacConkey agar containing ampicillin (100 mg/L) supplemented with rifampicin (1500 mg/L; Sigma Chemical Co.). The colonies grown on the selecting medium were selected, identified and tested for antimicrobial susceptibility by the VITEK2 compact system (bioMerieux, Hazelwood, MO, France).

### Transformation Experiment and Antimicrobial Susceptibility Test by VITEK2 Compact System

Plasmid DNAs isolated from the clinical No. 35 *E. Cloacae* strain were transformed by electroporation into *E. Coli* DH5Θ (Invitrogen, Auckland, New Zealand). The possible NDM-producing transformants were selected on Luria–Bertani agar supplemented with ampicillin (100 mg/L). The colonies grown on the selecting medium were selected, identified and tested for antimicrobial susceptibility by the VITEK2 compact system (bioMerieux, Hazelwood, MO, France).

### Plasmid Analysis

Plasmid DNAs isolated from the clinical No. 35 *E. Cloacae* strain (donor strain), *E. coli* EC600, representative transconjugant, *E. Coli* DH5Θ and representative transformant were obtained by the alkaline lysis method and were used as a template in PCR analyses with primers that are specific for *qnrA1*, *qnrS1*, *AAC(6′)-Ib*, *AAC(6′)-Ib-cr*, *bla*_DNM-1_ and *bla*_IMP-26_. The PCR products were purified and sequenced twice on both strands by Invitrogen (Invitrogen, Shanghai).

### PFGE Fingerprinting

Chromosomal DNA was digested with *Xba* I and subjected to pulsed-field gel electrophoresis (PFGE) as previously reported [24]. A lambda ladder (Bio-Rad, France) was used as the molecular weight marker. DNA macro restriction patterns were analyzed by visual inspection, based on the criteria of relatedness proposed by Tenover [25].

## Results

### Prevalence of the *QRD* Genes and their Associations with Carbapenemases and ESBLs Production

The *qnrA*, *qnrB*, *qnrS* and *aac(6′)-Ib-cr* genes were detected in 4 (11.4%), 8 (22.9%), 13 (37.1%) and 14(15.3%) of the isolates, respectively, including 2 with *qnrA1*, 2 with *qnrA2*, 2 with *qnrB2*, 6 with *qnrB6*, and 13 with *qnrS1*. The prevalence of any *QRD* genes was 68.6% (24/35).

The *qnrA*, *qnrB*, *qnrS* and *aac(6′)-Ib-cr* genes and any *QRD* genes were detected in 13.0% (3/23), 30.4% (7/23), 26.1% (6/23), 30.4% (7/23) and 60.9% (14/23) of the ESBL positive isolates, respectively. Interestingly, 10 of the 24 *QRD* positive isolates (41.7%) expressed the *bla_TEM-6_* gene.

Among the 9 carbapenemase-producers, 2 (22.2%) *qnrA*-positive, 4 (44.4%) *qnrB*-positive, 2 (22.2%) *qnrS*-positive, 3 (33.3%) *aac(6′)-Ib-cr*-positive, and 5 (55.5%) any *QRD*-positive strains were observed. Notably, while all the 3 *bla_KPC-2_* positive strains were shown not to express any *QRD* genes, 4 of the 5 *bla_IMP-8_* positive isolates (80.0%) expressed one or more *QRD* genes, and the only one strain co-expressing the *bla_IMP-26_* and *bla_NDM-1_* MBL genes was demonstrated to co-harbor *qnrA1*, *qnrS1* and *aac(6′)-Ib-cr*.

Moreover, the prevalence rates of *QRDs* among ESBL and carbapenemase co-producers and both of them non-producers were 66.7% (4/6) and 100.0% (9/9), respectively. Of note, only one was found in the ESBL- and carbapenemase+ group (Table 2).

**Table 2:** Relevant phenotypic and genotypic characteristics of the 35 carbapeneme non-susceptible (CNS) *Enterobacter cloacae* (*E. cloacae*) strains.

No.	MIC(mg/ml)		Drug resistance profiles	detection of resistance genes				PFGE pattern
	IMP	ETP		Carbape-nemase	ESBL	QRD	ARD	
1	2	16	CRO, CAZ, FEP, CIP, LEV, AK	*bla_IMP-8_*	-	-	*aac(6‘) -Ib*	A1
2	2	16	CRO, CAZ, FEP, CIP,LEV, GM, TOB, AK	*bla_IMP-8_*	*bla_TEM-6_*	*qnrB6*	*aac(6‘) -Ib*	A2
3	2	16	CRO, CAZ, FEP, CIP, LEV, GM, TOB, AK	*blaI_MP-8_*	*bla_TEM-6_, bla_CTX-M-_*	*qnrB6, aac(6′)-Ib-cr*	*aac(6‘) -Ib*	A1
4	0.25	4	CRO, CAZ, FEP, CIP, LEV, GM, TOB, AK	-	*bla_TEM-6_*	*-*	*armA, aac(6‘) -Ib*	E1
5	2	16	CRO, CAZ, FEP, CIP, LEV, GM, TOB, AK	*bla_IMP-8_*	*bla_TEM-6_*	*qnrB6*	*aac(6‘) -Ib*	A2
6	0.25	4	CRO, CAZ, FEP, CIP, LEV, GM, TOB, AK	*-*	*bla_TEM-6_*	*qnrA2*	*-*	K
7	8	8	CRO, CAZ, FEP, CIP, LEV, GM, TOB, AK	*-*	*-*	*qnrS1*	*aac(6‘)-Ib*	F
8	8	2	CRO, CAZ, FEP, GM, TOB, AK	*bla_IMP-8_*	*bla_TEM-6_*, *bla_SHV-12_*,*bla_CTX-M-14_*	*qnrA2, qnrB6, qnrS1, aac(6′)-Ib-cr*	*rmtB, aac(6‘) -Ib*	G
9	1	16	CRO, CAZ, FEP, CIP, LEV, GM, TOB, AK	*-*	*bla_TEM-6_*, *bla_SHV-12_*,*bla_CTX-M-3_*	*aac(6′)-Ib-cr, qnrS1*	*armA*	B1
10	1	16	CRO, CAZ, FEP, CIP, LEV, GM, TOB, AK	*-*	*bla_TEM-6_*, *bla_SHV-12_*,*bla_CTX-M-3_*	*aac(6′)-Ib-cr*	*armA*	B1
11	0.25	4	CRO, CAZ, FEP, CIP, LEV, GM, TOB, AK	*-*	*bla_TEM-6_*, *bla_CTX-M-3_*,*bla_CTX-M-14_*	*-*	*armA*	C2
12	0.25	4	CRO, CAZ, FEP, CIP, LEV, GM, TOB, AK	*-*	*bla_TEM-6_*, *bla_CTX-M-3_*,*bla_CTX-M-14_*	*aac(6′)-Ib-cr*	*armA, aac(6‘) -Ib*	C1
13	0.25	4	CRO, CAZ, FEP	*-*	*-*	*aac(6′)-Ib-cr*	*-*	J
14	0.25	2	CRO, CAZ, FEP, CIP, LEV, GM, TOB	*-*	*bla_TEM-6_*, *bla_SHV-12_*,*bla_CTX-M-3_*	*-*	*aac(6′) -Ib*	I
15	8	8	CRO, CAZ, FEP	*-*	*-*	*aac(6′)-Ib-cr*	*-*	H
16	0.25	4	CRO, CAZ, FEP, CIP, LEV, GM, TOB, AK	*-*	*bla_TEM-6_*, *bla_CTX-M-3_*	*qnrB6, aac(6′)-Ib-cr*	*armA*	D2
17	0.25	4	CRO, CAZ, FEP, CIP, LEV, GM, TOB, AK	*-*	*bla_TEM-6_*, *bla_CTX-M-3_*	*qnrB6, qnrS1*	*armA*	D1
18	1	16	CRO, CAZ, CIP, LEV, GM, TOB	*-*	*bla_SHV-12_*	*-*	*-*	B2
19	0.25	2	CRO, CAZ, FEP, CIP, LEV, GM, TOB, AK	*-*	*-*	*aac(6′)-Ib-cr*	*armA, aac(6‘) -Ib*	E2
20	2	16	CRO, CAZ, FEP, CIP, LEV, GM, TOB, AK	*-*	*-*	*qnrS1, aac(6′)-Ib-cr*	*armA, aac(6‘) -Ib*	E3
21	0.25	2	CRO, CAZ, FEP, CIP, LEV, GM, TOB, AK	*-*	*bla_SHV-12_*	*qnrS1*	*aac(6‘) -Ib*	E1
22	2	16	CRO, CAZ, FEP, CIP, LEV, GM, TOB, AK	*-*	*-*	*qnrS1, aac(6′)-Ib-cr*	*armA, aac(6‘) -Ib*	E3
23	16	16	CRO, FEP, CAZ, CIP, LEV, GM, TOB	*-*	*bla_SHV-12_*	*-*	*-*	L1
24	64	32	CRO, FEP, CAZ, CIP, LEV, GM, TOB	*bla_KPC-2_*	*bla_SHV-12_*	*-*	*-*	L2
25	32	32	CRO, FEP, CAZ, CIP, LEV, GM, TOB, AK	*bla_KPC-2_*	*bla_SHV-12_*, *bla_CTX-M-14_*	*-*	*aac(6‘) -Ib*	L1
26	2	16	CRO, FEP, CAZ, CIP, LEV, GM, TOB, AK	*-*	*bla_CTX-M-14_*	*qnrA1*	*-*	O
27	32	16	CRO, FEP, CAZ, CIP, LEV, GM, TOB	*bla_KPC-2_*	*-*	*-*	*-*	N
28	0.5	8	CRO, FEP, CAZ, CIP, LEV, GM, TOB, AK	*-*	*bla_CTX-M-14_*	*-*	*armA*	P
29	0.125	4	CRO, FEP, CAZ, CIP, LEV, GM, TOB, AK	*-*	*bla_CTX-M-14_*	*qnrB2, qnrS1*	*armA, aac(6‘) -Ib*	ND*
30	0.25	4	CRO, FEP, CAZ, CIP, LEV, GM, TOB, AK	*-*	*-*	*qnrB2, qnrS1*	*aac(6′) -Ib*	ND*
31	0.25	4	CRO, FEP, CAZ, CIP, LEV, GM, TOB	*-*	*bla_CTX-M-14_*	*-*	*aac(6′) -Ib*	M
32	0.125	4	CRO, FEP, CAZ, CIP, LEV	*-*	*bla_CTX-M-3_*	*qnrS1, aac(6′)-Ib-cr*	*aac(6′) -Ib*	Q
33	4	8	CRO, CAZ, CIP, LEV, GM, TOB, AK	*-*	*-*	*qnrS1*	*aac(6‘) -Ib*	E3
34	0.125	1	CRO, FEP, CAZ, CIP,LEV, GM, TOB, AK	*-*	*-*	*qnrS1, aac(6′)-Ib-cr*	*aac(6‘) -Ib*	E2
35	64	32	CRO, FEP, CAZ, CIP, LEV, GM,TOB	*bla_NDM-1,_ bla_MP-26_*	*-*	*qnrA1, qnrS1,* *aac(6′)-Ib-cr*	*aac(6‘) -Ib*	O

* ND: not determined.

### Prevalence of the *ARD* Genes and their Associations with Carbapenemases and ESBLs Production

The *aac(6′)-Ib*, *armA* and *rmtB* genes were detected in 21 (60.0%), 12 (34.3%), and 1 (2.9%) of the 35 isolates, respectively. Since 6 isolates carried both *aac(6′)-Ib* and *armA*, one isolate carried both *aac(6′)-Ib* and *rmtB*, the prevalence of any *ARD* genes was 27 (87.1%) of all isolates. The *aac(6′)-Ib*, *armA* and *rmtB* genes were detected in 52.2% (12/23), 39.1% (9/23), and 4.3% (1/23) of the ESBLs positive isolates, respectively. Of note, 17 (63.0%) strains of the 27 *ARD* positive isolates expressed *bla_TEM-6_* or *bla_CTX-M-like_* genes.

Interestingly, while only 1 of the 3 *bla_KPC-2_* positive strains was shown to express the *aac(6′)-Ib* gene, all the 5 *bla_IMP-8_* positive isolates (100.0%) expressed the *aac(6′)-Ib* gene, and the only one strain co-expressing the *bla_IMP-26_* and *bla_NDM-1_* MBL genes was also demonstrated to harbor the *aac(6′)-Ib* gene, that is to say, 7 carbapenemase positive strains co-expressed the *aac(6′)-Ib* gene.

Moreover, the prevalence rates of *ARD* among ESBL and carbapenemase co-producers and both of them non-producers were 83.3% (5/6) and 77.8% (7/9), respectively. Notably, most of the ESBL+ and carbapenemase- strains (13/17, 76.5%) were shown to be *ARD*-positive (Table 2).

### Plasmid Analysis

Firstly, using *E. coli* EC600 (rifampicin resistant) as the recipient, a conjugation experiment was performed to investigate whether the drug resistance genes identified in the clinical No. 35 *E. Cloacae* strain were located on plasmids and whether the transfer of these genes contributed to the reduced susceptibility of the recipient *E.* *coli* EC600 towards antibiotics. The transconjugants showed a multidrug resistance phenotype that included resistance to ampicillin, ampicillin/sulbactam, piperacillin/tazobactam, cefazolin, cefotetan, ceftazidime, ceftriaxone, ertapenem, imipenem and ciprofloxacin, intermediate susceptible to cefepime and levofloxacin. However, all the transconjugants were susceptible to aztreonam, amikacin, gentamicin and tobramycin (Table 3). For the β-lactam antibiotics or β-lactam/β-lactamase inhibitor combinations, the transconjugants showed ≥2-, 8-, 32-, 16-, 16-, 64-, 64-, 16-, 16- and 16-fold increases in the MICs of ampicillin, ampicillin/sulbactam, piperacillin/tazobactam, cefazolin, cefotetan, ceftriaxone, ceftazidime,cefepime, ertapenem and imipenem, respectively, when compared with the recipient *E. coli* EC600 strain. For quinolone antibiotics, the transconjugants showed ≥16- and 8-fold increases in the MICs of ciprofloxacin and levofloxacin, respectively, when compared with the recipient strain. With regard to the MICs of aztreonam, amikacin, gentamicin, and tobramycin, they showed no increases. More notably, eight transconjugants were randomly chosen and detected to harbour *qnrA1*, *qnrS1*, *aac(6′)-Ib-cr*, *bla*_DNM-1_ and *bla*_IMP-26_ simultaneously, while *aac(6′)-Ib* was not detected in all the transconjugants (Table 4).

**Table 3:** MICs of antibiotics for the clinical No. 35 *E. Cloacae* strain (donor strain), *E. coli* EC600, representative transconjugant, *E. Coli* DH5? and representative transformant.

antibiotics	MIC (µg/ml)				
	EC No.35	EC600	Transconjugant*	DH5?	Transformant*
AMP	≥32	16	≥32	≤2	≥32
AMS	≥32/16	4/2	≥32/16	≤2/1	≥32/16
PTZ	≥128/4	≤4/0.125	≥128/4	≤4/0.125	64/2
CFZ	≥64	≤4	≥64	≤4	≥64
CTT	≥64	≤4	≥64	≤4	32
CRO	≥64	≤1	≥64	≤1	≥64
CAZ	≥64	≤1	≥64	≤1	≥64
FEP	≥64	≤1	16	≤1	8
AZT	≥64	≤1	≤1	≤1	≤1
IMP	≥16	≤1	≥16	≤1	≥16
ETP	≥8	≤0.5	≥8	≤0.5	≥8
CIP	≥4	≤0.25	≥4	≤0.25	0.5
LEV	≥8	0.5	4	≤0.25	1
GM	≥16	≤1	≤1	≤1	≤1
TOB	8	≤1	≤1	≤1	≤1
AK	≤2	≤2	≤2	≤2	≤2

* Eight representative transconjugants and transformants were respectively collected for antibiotic susceptibility test by the AST GN-13 card, and the representative results were shown.

Abbreviations used: ampicillin (AMP), ampicillin/sulbactam (AMS), piperacillin/tazobactam (PTZ), cefazolin (CFZ), cefotetan (CTT), ceftazidime (CAZ), ceftriaxone (CRO), cefepime (FEP), aztreonam (AZT), ertapenem (ETP), imipenem (IMP), ciprofloxacin (CIP), levofloxacin (LEV), amikacin (AK), gentamicin (GM), tobramycin (TOB).

**Table 4:** Drug resistance genes detected from the clinical No. 35 *E. Cloacae* strain (donor strain), *E. coli* EC600, transconjugants, *E. Coli* DH5? and transformants using their plasmids as PCR templates.

Genes	Stains				
	EC No.35	EC600	transconjugant	DH5?	transformant
*bla*_IMP-26_	•		•		•
*bla*_DNM-1_	•		•		•
*qnrA1*	•		•		
*qnrS1*	•		•		•
*AAC(6′)-Ib*	•		•		
*AAC(6′)-Ib-cr*	•				

•denotes positive.

Secondly, using *E. coli* DH5Θ as the recipient, a transformation experiment was performed to further investigate whether these genes were located on plasmids and whether the transfer of these genes contributed to the reduced susceptibility of the recipient *E. coli* DH5Θ towards antibiotics. The *E. Coli* DH5Θ transformants showed resistance to ampicillin/inhibitor combinations, broad-spectrum cephalosporins and carbapenems (Table 3). Similar to those of the transconjugants, all the transformants were also susceptible to aztreonam, amikacin, gentamicin, and tobramycin. However, on the other hand, although the transformants were still susceptible to ciprofloxacin and levofloxacin, they showed ≥2- and 4-fold increases in the MICs of ciprofloxacin and levofloxacin, respectively, when compared with the recipient *E. Coli* DH5Θ strain. More notably, transformation assays allowed to transfer the *qnrS1*, *bla*_DNM-1_ and *bla*_IMP-26_ gene simultaneously in all the transformants tested (Table 4).

### Molecular Epidemiology

**Figure 1:**
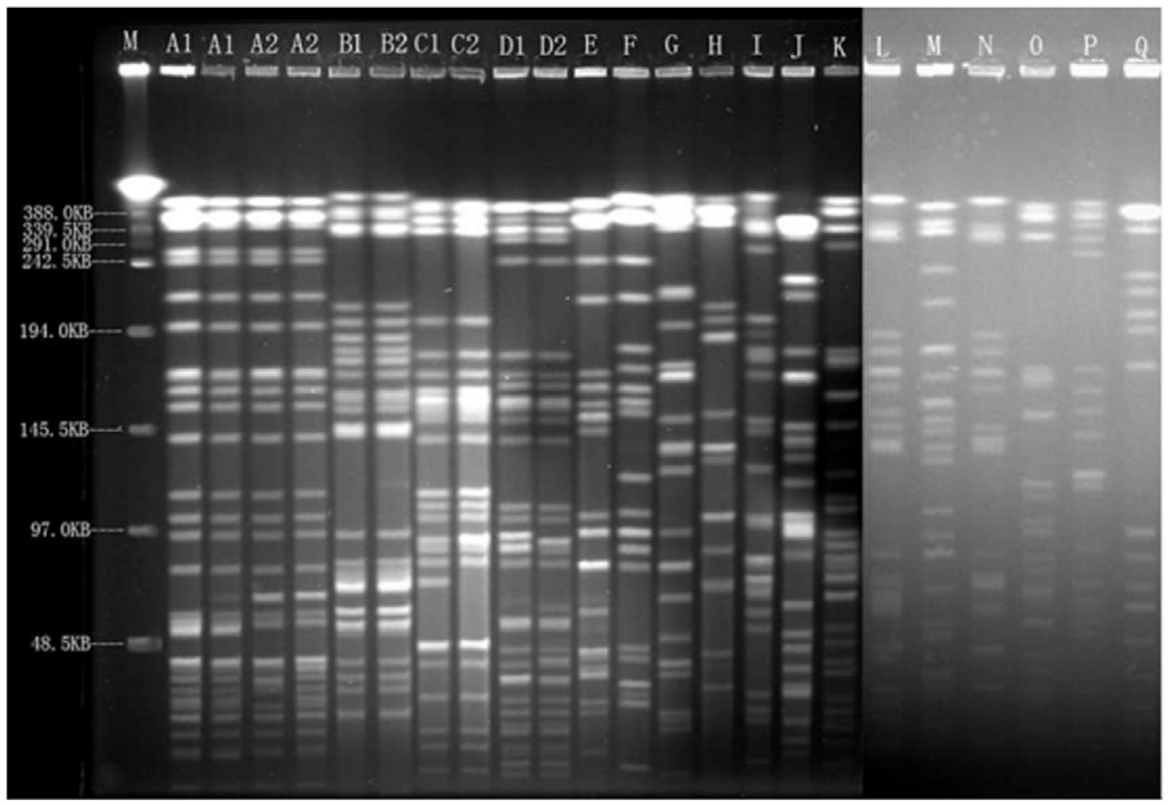
PFGE patterns of 35 CNS *E. cloacae* isolates. PFGE fingerprinting was performed according to the “methods” section. Chromosomal DNA restriction patterns were interpreted by the Tenover's criteria. M: Lambda DNA Ladder; A1-Q: representative isolates with different genotype from A to Q.

Seventeen *Xba* I patterns, named A to Q, were found among the 35 *E. cloacae* isolates. Between Sep 2009 and Feb 2011, the major epidemic pattern A comprised of 4 isolates genetically related to subtypes A1 and A2. All of the pattern A isolates were *bla_IMP-8_* positive and were all collected from different wards. However, the fifth *bla_IMP-8_* positive isolate, identified nine months after the fourth IMP-8 producer was isolated, was collected in another ward and was found to be totally different from pattern A and were grouped into pattern G. The isolates collected from Sep 2011 to Feb 2012 were categorized into eight clusters. The major epidemic pattern E comprised of 6 isolates genetically related to subtypes E1, E2 and E3 (Figure 1 and Table 2). These data indicate that the high prevalence of CNS *E. cloacae* isolates was not caused by clonal dissemination.

## Discussion

In the present study, the prevalence of the *PMQRs* and *PMARs* among the 35 CNS *E. cloacae* isolates in a Chinese teaching hospital was characterized and their molecular epidemiological characteristics were also achieved. Of the 35 isolates, 9 harbored carbapenemase genes, 23 carried ESBLs, 24 were *QRD* positive and 27 were *ARD*-producers. Among the 5 *bla_IMP-8_* positive strains, 4 contained *QRD* genes, and 5 harbored *ARD* genes. Among the 23 ESBLs positive isolates, 14 were QRD positive and 18 were ARD positive. Molecular typing by PFGE revealed genetic diversity among the 35 isolates, indicating that the CNS *E. cloacae* isolates were epidemiologically unrelated. Of note, multiple resistant genes were found to be co-expressed in the same CNS *E. cloacae* isolates.

The increasing frequency of quinolone resistance in *Enterobacteriaceae* was associated with an increasing prevalence of *PMQR* genes, and this change involved an increase in the diversity of the *PMQR* genes and also an increase in the prevalence of the mutations in *gyrA*, *parC*, or both in *PMQR*-positive strains but not *PMQR*-negative strains [26]. The present study demonstrated high prevalence (24/35, 68.6%) of *QRDs* among CNS *E. cloacae* isolates in a Chinese teaching hospital. The association of *QRD* with *bla_IMP-8_* or ESBLs should be investigated further.

Three common plasmid-borne *qnr* genes, *qnrA*, *qnrB* and *qnrS* were detected, with *qnrS* (37.1%, 13/35) being the most common among the three. Moreover, the low rates of *qnrA* have been observed in most surveillance studies [16], while in some countries, *qnrA* were more common [27]. Interestingly, isolate 8 co-carrying *qnrA2*, *qnrB6*, *qnrS1* and *aac(6′)-Ib-cr*, was found to be sensitive to both CIP and LEV. Q*nr* determinants alone may not confer resistance to quinolones, but they can supplement other quinolone resistance mechanisms. In our study, one isolate (isolate 8) carrying *qnrA2*, *qnrB6*, *aac(6′)-Ib-cr* and *qnrS1* and 2 isolates (isolate 13 and 15) harboring *aac(6′)-Ib-cr* were classified as Ciprofloxacin (CIP) and levofloxacin (LEV) susceptible by CLSI 2012 criteria. Thus, treatment with a fluoroquinolone might easily select for resistant strains, and it will be interesting to find out whether the existence of the *qnr* genes is sufficient to facilitate the selection of mutants with higher levels of quinolone resistance. Most importantly, this is the first report of the co-expression of *qnrA1* and q*nrS1* with *bla_DNM-1_* and *bla_IMP-26_* carbapenemases in *E. cloacae*.

Many carbapenemase-producing *Enterobacteriaceae* are highly multiresistant, but may remain susceptible to one or more aminoglycosides [28]. In the present study, 11 (31.4%) strains were found to be sensitive to at least one of the aminoglycosides tested. Three common *ARD* genes, *aac(6′)-Ib*, *armA* and *rmtB* were detected, with *aac(6′)-Ib* being the most common (60.0%, 21/35) among the three. The extremely high prevalence (27/35, 77.1%) of *ARD* among CNS *E. Cloacae* in our hospital may be due mainly to the intrahospital spread of a few clones and the dissemination of plasmids containing both *aac(6′)-Ib* or *armA* and ESBLs, presumably by means of transposons, insertion sequences or recombination. The semisynthetic aminoglycoside amikacin (AK) is very useful in the treatment of multiresistant infections because only a limited number of modifying enzymes, such as AAC(6′)-I-type acetyltransferases, are able to inactivate it. Unfortunately, the rise in multiresistant strains harboring *aac(6′)-Ib* has seriously limited the successful use of aminoglycosides including AK [29]. Notably, although all the 3 *bla*_KPC-2_ positive carbapenem-resistant *E. cloacae* strains included in this study were found not to carry any *QRDs*, and only one showed the co-expression of one kind of *ARD*, *aac(6′)-Ib*, they were shown to be resistant to both quinolones (CIP and LEV) and aminoglycosides (GM and TOB), and notably, the only one co-harboring *bla_KPC-2_* and *aac(6′)-Ib* demonstrated to be resistant to GM, TOB and AK, while the other two without *aac(6′)-Ib* were AK-susceptible.

The emergence and spread of carbapenem-resistant *Enterobacteriaceae* in the world are a major concern. Q Wu et al. [30] demonstrated 5 isolates of *E. cloacae* from Shanghai, China, that were resistant to all clinically available antimicrobial agents co-expressing *bla_KPC-2_*, *bla_SHV-12_*, *bla_CTX-M-14_* and *armA* 16S rRNA methylase. We reported for the first time an *E. cloacae* strain co-harboring *bla_KPC-2_*, *bla_SHV-12_*, *bla_CTX-M-14_* and aac(6′)-Ib that was also resistant to all clinically available antimicrobial agents from Chongqing.

To our knowledge, this is the first description of the coexistence of the *qnrA1*, *qnrS1*, *aac(6′)-Ib*, *aac(6′)-Ib-cr*, *bla*_DNM-1_ and *bla*_IMP-26_ resistance genes in one enterobacterial strain. The association of these resistance determinants is worrisome, because it may facilitate the selection of high-level multidrug-resistant strains in some communities and this process may be promoted by the co-selection of various antimicrobial agents under subinhibitory concentrations. There is a great need to obtain more detailed knowledge on the association of various resistance genes in enterobacterial strain, and more studies should be carried out in this field. We suggest that an effective surveillance and strict infection control strategies should be implemented soon to prevent potential outbreaks of nosocomial infections by such pathogens in China.

In conclusion, *QRD* and *ARD* genes were highly prevalent among the CNS *E. cloacae* isolates, and multiple resistant genes were co-expressed in the same isolates. Most importantly, the CIP- and LEV-susceptible CNS *E. cloacae* isolate co-expressing *bla_IMP-8_*, *bla_TEM-6_*, *bla_SHV-12_*, *bla_CTX-M-14_*, *qnrA2*, *qnrB6*, *qnrS1*, *aac(6′)-Ib-cr*, *rmtB* and *aac(6′)-Ib* and the CNS *E. cloacae* isolate co-expressing *qnrA1*, *qnrS1*, *AAC(6′)-Ib*, *AAC(6′)-Ib-cr*, *bla*_DNM-1_ and *bla*_IMP-26_ were first reported to the best of our knowledge. Southern blot experiments are currently underway to reveal the the possible co-existence of *bla_NDM-1_*, *bla_IMP-26_* and *qnrS1* on the same transferred plasmid in the clinical No. 35 *E. Cloacae* strain.
